# COVID-19: Guidelines for pharmacists in South Africa

**DOI:** 10.4102/sajid.v35i1.206

**Published:** 2020-06-10

**Authors:** Natalie Schellack, Monique Coetzee, Gustav Schellack, Michelle Gijzelaar, Zeenat Hassim, Marnus Milne, Elmien Bronkhorst, Neelaveni Padayachee, Nirasha Singh, Sonya Kolman, Andrew L. Gray

**Affiliations:** 1 School of Pharmacy, Faculty of Health Sciences, Sefako Makgatho Health Sciences University, Pretoria, South Africa; 2 Pharmacy Services, Mediclinic Southern Africa, Stellenbosch, South Africa; 3 Pharmaceutical Industry, Pretoria , South Africa; 4 Pharmacy Practice Department, Life Healthcare, Johannesburg, South Africa; 5 Pharmacy Department, Dr George Mukhari Academic Hospital, Pretoria, South Africa; 6 Department of Pharmacy and Pharmacology, Faculty of Health Sciences, University of the Witwatersrand, Johannesburg, South Africa; 7 Clinical Pharmacy Services, Netcare Limited, Johannesburg, South Africa; 8 Pharmacy, Nelson Mandela Children’s Hospital, Johannesburg, South Africa; 9 Department of Pharmacy, School of Therapeutic Sciences, Faculty of Health Sciences, University of the Witwatersrand, Johannesburg, South Africa; 10 Division of Pharmacology, Discipline of Pharmaceutical Sciences, University of KwaZulu-Natal, Durban, South Africa

**Keywords:** novel coronavirus (2019-nCoV), corona virus disease, pandemic, COVID-19, SARS-CoV, MERS-CoV, SARS-CoV-2, spill-over, chloroquine, hydroxychloroquine

## Abstract

Since the outbreak of COVID-19, and its declaration as a pandemic by the World Health Organization (WHO), the reliance on pharmacists as one of the first points of contact within the healthcare system has been highlighted. This evidence-based review is aimed at providing guidance for pharmacists in community, hospital and other settings in South Africa, on the management of patients with suspected or confirmed coronavirus disease 2019, or COVID-19. The situation is rapidly evolving, and new evidence continues to emerge on a daily basis. This guidance document takes into account and includes newly available evidence and recommendations, particularly around the following aspects relating to COVID-19:

- Epidemiology
- The virus, its modes of transmission and incubation period
- Symptom identification, including the differentiation between influenza, allergic rhinitis, sinusitis and COVID-19
- Social media myths and misinformation
- Treatment guidelines and medicines that may need to be kept in stock
- Treatment and prevention options, including an update on vaccine development
- The case for and against the use of NSAIDs, ACE-inhibitors and angiotensin receptor blockers (ARBs) in patients with COVID-19
- Interventions and patient counselling by the pharmacist.

It is critical, though, that pharmacists access the most recent and authoritative information to guide their practice. Key websites that can be relied upon are:

- World Health Organization (WHO): https://www.who.int/emergencies/diseases/novel-coronavirus-2019
- National Institute for Communicable Diseases (NICD): https://www.nicd.ac.za/diseases-a-z-index/covid-19/
- National Department of Health (NDoH): http://www.health.gov.za/index.php/outbreaks/145-corona-virus-outbreak/465-corona-virus-outbreak; https://sacoronavirus.co.za/

Updated on 10 May 2020. Writing Group of the South African Society of Clinical Pharmacy (SASOCP): COVID-19 Guidelines for Pharmacists in South Africa.

## Introduction and brief epidemiology

Coronavirus disease 2019 (COVID-19) has been declared a pandemic, meaning that there is now a global spread. Coronaviruses are typically associated with the common cold, and therefore with mild forms of respiratory (and sometimes also gastrointestinal) illness.^1,2^

However, two novel coronaviruses have caused more severe respiratory illness in the past, namely^1,2^:

- The severe acute respiratory syndrome coronavirus, or SARS-CoV, which was first identified in China, in 2003, and
- The Middle East respiratory syndrome coronavirus, or MERS-CoV, which was first identified in Saudi Arabia in 2012.

The current outbreak of a novel coronavirus (first dubbed 2019-nCoV), began in Wuhan, China, and was reported to the local Country Office of the World Health Organization (WHO) on 31 December 2019, with a cluster of pneumonia cases. These cases were soon linked to a previously unknown virus, which has since been identified and named as SARS-CoV-2. The disease caused by this novel coronavirus is called COVID-19.^1,2^

The initial cluster of pneumonia cases in Wuhan were all associated with a local marketplace, which sells seafood and live animals. Like the two previous examples, this novel coronavirus is assumed to be zoonotic (i.e. involving a species change, or so-called spill-over, from an animal reservoir). SARS-CoV is believed to have originated from a virus affecting civet cats, and MERS-CoV from dromedary camels.^1,2^

A specific reverse transcriptase-polymerase chain reaction (RT-PCR) test is currently required to make a definitive diagnosis, and current treatment is mostly aimed at symptomatic relief and the support of vital functions. Serological tests aimed at detected antibodies (IgM/IgG) against SARS-CoV-2 have also been developed but cannot be relied on for diagnosing acute infections.^1,2^ The Food and Drug Administration (FDA) recently approved Xpert^®^ Xpress SARS-CoV-2 for rapid confirmation on existing GeneXpert diagnostic platforms (refer to: https://www.fda.gov/media/136314/download).

Note:

- The virus = SARS-CoV-2 (Severe acute respiratory syndrome coronavirus-2)
- The disease = COVID-19 (Coronavirus disease-2019)

South Africa has a population of more than 59 million. There are currently around 7 million people living with the human immunodeficiency virus (HIV), of which more than 5.1 million are on antiretroviral therapy (ART). In addition, there are about 400 000 people living with active tuberculosis (TB), 4 million with diabetes and 14 million with various types of cancer.^2^

HIV, TB, cancer and diabetes are all major conditions that can compromise the immune system. As suggested by the WHO, such patients are expected to be at higher risk for developing more severe forms of COVID-19. Older adults and those with severe cardiovascular or respiratory disease are also expected to be at higher risk.^1^

It is expected that the total number of infected individuals in South Africa will continue to rise over the coming months, before the plateau in the epidemic curve will be experienced. Public and private laboratories are now fully equipped to test for SARS-CoV-2, the causative pathogen of COVID-19.^2^ In their efforts to expand and streamline testing of COVID-19, the National Institute for Communicable Diseases (NICD) has decided that doctors no longer need approval from them to test for the virus but should still communicate with their local laboratory experts in this regard. The laboratories will conduct the diagnostic test, provided that the case definition is applied, and the required supporting documents accompany the sample.^2^ More information in this regard may be obtained from the NICD’s website: https://www.nicd.ac.za/diseases-a-z-index/covid-19/covid-19-guidelines.

COVID-19 has predominantly affected the adult population, and in particular the elderly population, with children having less severe clinical manifestations.^3^ Recent studies have shown that the human receptor for the virus, the angiotensin-converting enzyme 2 (ACE2) receptor, is not expressed to the same extent in children, perhaps making them less likely to contract COVID-19.^3,4^ Additionally, children most likely have higher antibody levels compared to adults, because they are exposed to respiratory infections more often.^3^ Nevertheless, Dong et al. also showed that there was vulnerability amongst the infant population (<1 year of age) with 10.6% presenting with severe and critical cases compared to 7.3% in the age group of one to five years.^3^ However, some children have presented with severe and fatal disease.

## Symptoms, identification and transmission of COVID-19

The clinical presentation of patients that contract the virus include, in the prodromal phase, fever, dry cough and malaise and possibly a sore throat, with the latter being seen more readily in milder cases than in severe disease.^5,6^ Around 50% of patients may develop severe dyspnoea and some may even require mechanical ventilator support.^5,7^ Severe interstitial pneumonia may occur as a complication in up to 15% of patients. This, in turn, can lead to acute respiratory distress syndrome (ARDS), multi-organ failure (including acute renal failure, disseminated intravascular coagulation, or DIC), and death.^8^

In addition, COVID-19 patients have an increased risk of developing deep vein thrombosis (DVT) and pulmonary embolism (PE). COVID-19 appears to induce a disease-specific hypercoagulable state, characterised by cytokine-mediated diffuse microvascular damage, and in some cases, reactive thrombocytosis. The risk of DVT and PE is further complicated by the presence of obesity, advanced age and the immobility that results from hospitalisation. Standard DVT and PE precautions, prophylaxis and treatment guidelines should be applied where indicated.^8^

The incubation period of the virus is reported to be around 5 days, although it may be as long as 12.5 to 14 days. Currently it is still unclear when transmission of the virus happens and although the majority of secondary cases come from symptomatic individuals, some reported cases suggest that transmission during the asymptomatic phase may potentially be possible.^7^ The mode of transmission is considered to be respiratory, through respiratory droplets via coughing or sneezing (or even speaking) and other contact routes. However, such particles may remain present on surfaces like glass, plastic and steel for up to 4 days.^9^

## Social media myths and misinformation regarding COVID-19

As the COVID-19 pandemic grows, so does misinformation regarding the virus. Social media have created a panic amongst all age groups. Whilst these platforms are a possible means of obtaining information, it is important to consider the source and credibility of information posted on these forums; social media allows any information to be posted or accessed, regardless of its authenticity.

Factual information is a vital way to protect oneself against the disease and the pharmacist can play a critical role in countering misinformation by educating members of the public. Some of the myths and misinformation being spread via social media platforms are included in Table 1, and factual responses are indicated.

**TABLE 1:** Examples of myths that are appearing viral on social media and the facts that may assist in countering them (based on information provided by the World Health Organization).^10^

Myths†	Facts†
The virus can be killed by cold weather and snow.	Our body temperature remains in the range of 36.5°C to 37°C, regardless of the ambient temperature or weather. Similarly, the virus is not more or less likely to be transmitted in areas with hot and humid climates either.
Mosquitoes can transmit the virus.	The virus is not transmitted through blood. It is a respiratory virus that is transmitted through droplets from an infected person (during coughing, sneezing, or even when speaking).
Alcohol and chlorine sprayed all over the body can kill the virus in an infected person.	Alcohol and chlorine cannot kill the virus once it has already entered the body. These substances can be irritating or even harmful to the mucous membranes. Under certain recommendations they may, however, be used to disinfect surfaces. Hand sanitisers should contain 70% alcohol.
Pneumococcal and *Haemophilus influenza* type B (Hib) vaccines (used as protection against bacterial pneumonia) provide protection against the new coronavirus.	SARS-CoV-2 is a new virus, and the pathogens mentioned here are bacteria. A vaccine has not yet been developed against the coronavirus, but researchers are currently working on a range of potential options in this regard.
Eating garlic will prevent a person from contracting the virus.	There is no evidence that eating garlic can protect a person from the coronavirus.
Only older people are susceptible to infection with the new coronavirus.	The virus can affect people of all age groups; however, older people, and people with pre-existing medical conditions (e.g. asthma, heart disease or diabetes mellitus) are more susceptible to becoming severely ill with the virus.
Antibiotics can treat infected patients.	Antibiotics are mostly antibacterial agents. The coronavirus is not a bacterium. Thus, antibiotics will not be effective in treating the primary viral infection. However, patients who have the coronavirus with a bacterial co-infection, may need to receive antibiotics.
Wearing a mask can prevent a person from contracting the virus.	Masks are only effective as a means of protection when used correctly and in conjunction with other preventative measures (such as effective hand-washing or sanitising, and social distancing). Wearing a mask however does not negate the importance of proper hand-washing and social distancing.
Wearing gloves can prevent a person from contracting the virus.	Other coronaviruses have been shown to survive on latex or rubber gloves for up to 8 h. Also, gloves may prevent people from washing their hands. The WHO therefore recommends that people wash their hands regularly with soap and water, or spray with an alcohol-based hand sanitiser to protect themselves from contracting the virus.

† , Visit the World Health Organization (WHO) website for more information: https://www.who.int/emergencies/diseases/novel-coronavirus-2019/advice-for-public/when-and-how-to-use-masks

## Preventative measures and information to the public and pharmacists

**FIGURE 1:**
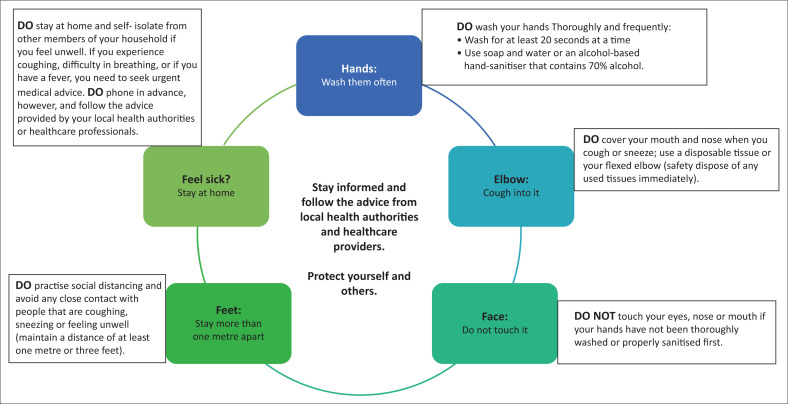
The most important preventative measures in the fight against COVID-19.^11^

**FIGURE 2:**
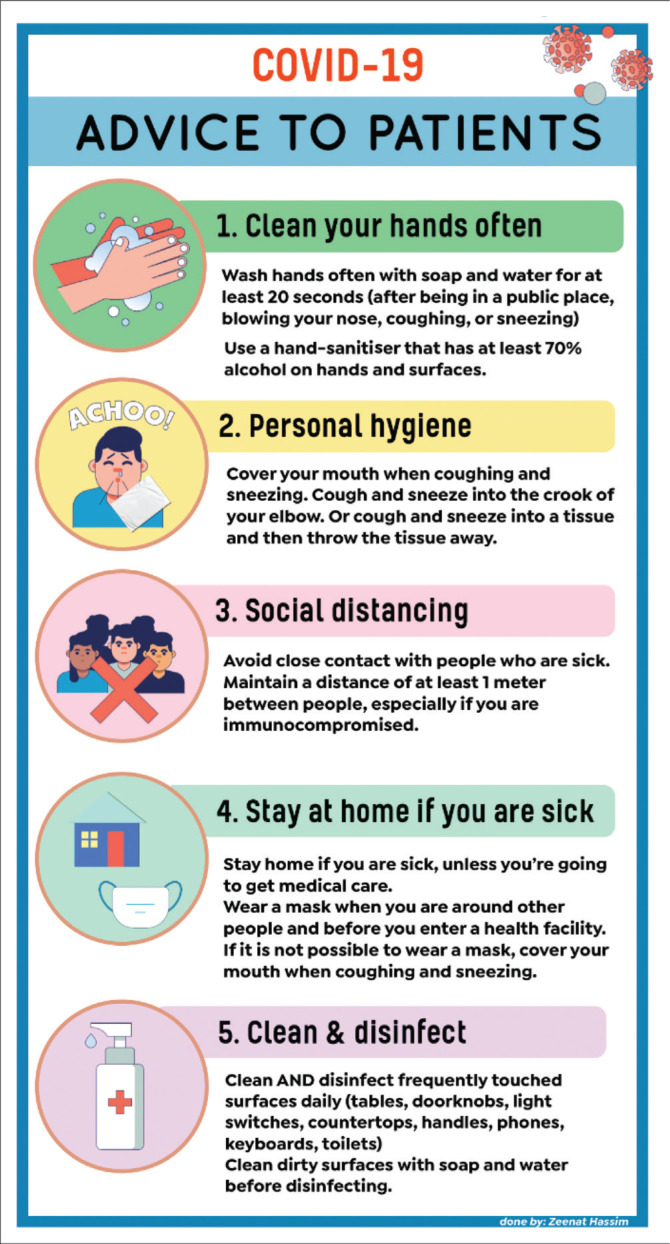
Advice to be given to patients and members of the public.^1,12,13^

**FIGURE 3:**
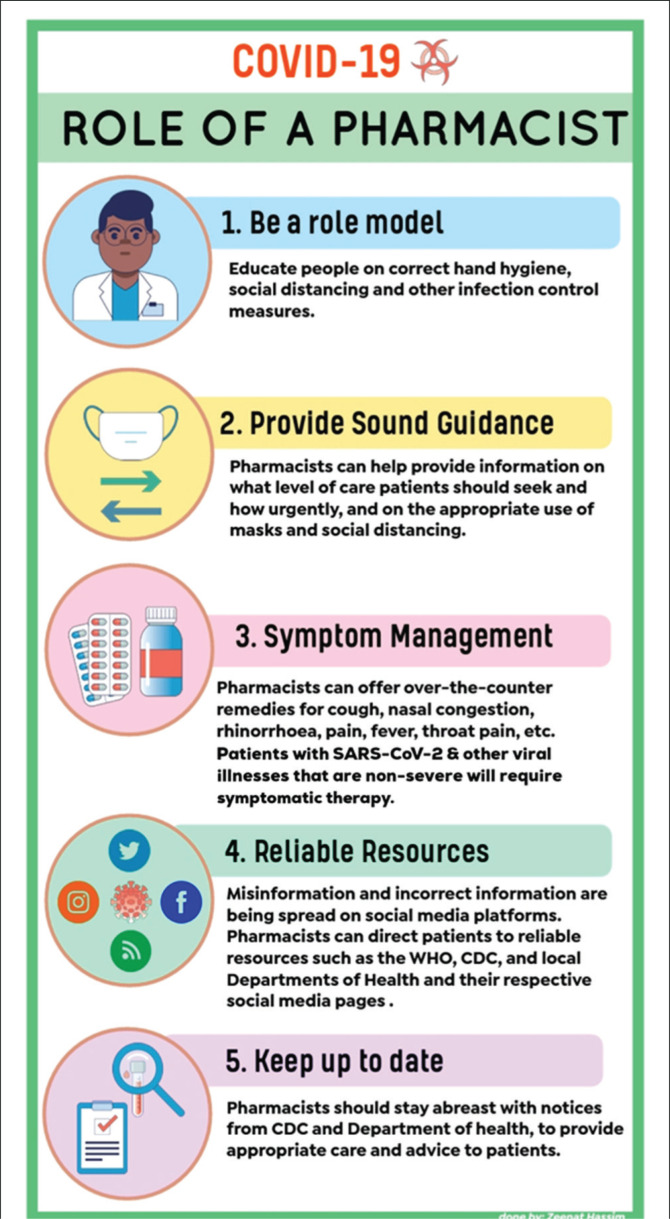
A basic summary of the role of the pharmacist in the management of COVID-19.^1,12,13^

The most important measures in trying to contain the spread of COVID-19 have been summarised in Figure 1.^11^ Figure 2 illustrates additional aspects pertaining to preventative measures that need to be observed by all members of society, and Figure 3 summarises the role that the pharmacist can play in this regard.^1,12,13^

## General treatment principles

**FIGURE 4:**
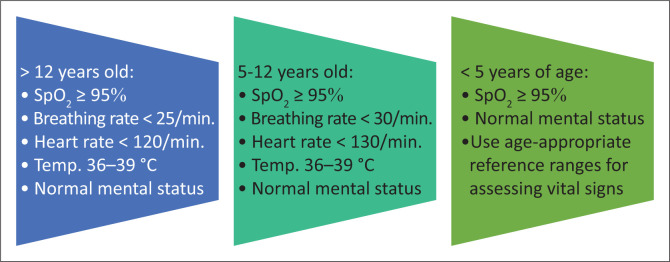
The criteria for mild COVID-19 according to the latest NDoH/NICD guidelines.^14^

At the time of writing these guidelines, the National Department of Health (NDoH) and the NICD in South Africa recommended the following^14^:

- The rapid triage of new cases to identify those with moderate, severe or critical illness. The criteria for mild disease are listed in Figure 4. Only patients with mild disease may be considered for isolation and management at home (i.e. with safe and effective self-isolation), and only if and when the required conditions, which have been outlined by the NDoH and NICD, have been met. A suitable patient information leaflet may also be handed to such patients. If their condition should worsen, these patients should seek follow-up medical attention as a matter of priority.^14^
- The treatment of confirmed COVID-19 cases is generally supportive and includes adequate oxygenation, conservative fluid management and antipyretic therapy.^1,14,15^
- Patients with moderate, severe or critical disease will need to be hospitalised for further management. The administration of supplemental oxygen therapy to patients with low peripheral oxygen saturation (SpO_2_) is of vital importance. The target SpO_2_ values are as follows^14^:
  ■ Adults in general: ≥ 90%
  ■ Pregnant women: ≥ 92%
  ■ Children: ≥ 92%
  ■ Children with respiratory distress or critical illness: ≥ 94%.
- Systemic corticosteroids should not be used routinely, unless indicated for another reason.^14,15^ However, there is emerging evidence to suggest that inhaled corticosteroids (ICS) may be beneficial in managing viral infections, specifically those that are caused by the coronavirus.^16^
- The enrolment of patients with COVID-19 in clinical trials to enable optimal contributions towards the much-needed pool of scientific evidence in this regard.^14^ Numerous clinical trials are currently under way around the globe, including trials in South Africa. More information about clinical trials that are currently in the process of recruiting patients, or that are presently underway, may be found at: www.clinicaltrials.gov” www.clinicaltrials.gov.

We currently do not have any scientific evidence from randomised, controlled trials (RCTs) to enable specific treatment recommendations for patients with suspected or confirmed COVID-19. There is also no evidence yet to support the use of any drug or vaccine to effectively prevent COVID-19 infection.^14^

Patients diagnosed with COVID-19 and that are at high risk for poor outcomes, including ARDS and death, are those that meet any of the criteria that are outlined in Table 2. Note that these guidelines are subject to change due to the dynamic nature of the current situation.

**TABLE 2:** Criteria for determining which patients are at high risk for poor outcomes.^16^

Patients that meet *any* of the following criteria	Variable
Age	≥ 65 years
Any of the following medical conditions	Cardiovascular disease, excluding hypertension as the sole cardiovascular diagnosis Diabetes mellitus with an A1c level > 7.5% Chronic pulmonary diseases, including asthma End-stage renal disease Advanced liver disease Blood disorders (e.g. sickle cell disease) Neurological or neurodevelopmental disorders Post-solid organ transplantation, on immunosuppressive therapy Use of biologic agents for immunosuppression Undergoing treatment with chemotherapy or immunotherapies for malignancy Within 1 year following a bone marrow transplant Undergoing treatment for graft-versus-host disease HIV infection, with a CD4 cell count < 200 copies/mm^3^.
Any of the following clinical findings	Oxygen saturation (SaO_2_) < 94% on room air; < 90% if there are known chronic hypoxic conditions present, or if the patient is receiving chronic supplemental oxygen Breathing rate > 24 breaths/min.
D-dimer level > 1 µg/mL in patients with respiratory illness.	-
Any hospitalised patient that develops any of the aforementioned medical conditions or clinical findings.	-

*Source*: Writing Group of the Johns Hopkins University and Johns Hopkins Hospital COVID-19 Treatment Guidance Working Group. JHMI clinical guidance for available pharmacologic therapies for COVID-19 [homepage on the Internet]. [cited 2020 Mar 21]. Available from: https://www.hopkinsguides.com/hopkins/ub?cmd=repview&type=479-1116&name=4_538747_PDF

### The case for chloroquine and hydroxychloroquine^14,15,16,17,18,19,20^

Pharmacists and prescribing clinicians and patients should be aware that specific drug efficacy for COVID-19 is unclear and still under investigation. The current NDoH/NICD guidelines do not recommend the use of chloroquine (CQ)/hydroxychloroquine (HCQ), due to insufficient evidence, in the treatment of patients with suspected or confirmed COVID-19.

There is very limited evidence that treatment with CQ and HCQ may result in a more rapid reduction in viral shedding and therefore may be associated with improved clinical outcomes. If CQ and HCQ do have clinically significant antiviral activity, then, based on experience with other acute viral infections, it is likely that they will be more effective when initiated as soon as possible. The use of HCQ will require Section 21 authorisation for named-patient access in South Africa.

Chloroquine is in short supply due to recent stockpiling and should be used in hospitalised patients only (for close monitoring purposes) and those who are at high risk for poor outcomes (as defined elsewhere).

The therapeutic index of CQ is quite narrow (especially in terms of its cardiotoxicity and resultant arrhythmias), requiring special caution when used at higher cumulative dosages.

Patients receiving high dosages, as part of COVID-19 management, need to be closely monitored for signs and symptoms of toxicity, such as headache, altered mental state, vertigo, visual impairment, convulsions, cardiac arrhythmias, dyspnoea, nausea and vomiting. The following contra-indications also need to be considered prior to the initiation of therapy: QTc-prolongation (> 500 ms), myasthenia gravis, porphyria, retinal pathology, epilepsy and potentially serious drug–drug interactions. Also note that the combination of hydroxychloroquine and azithromycin (HCQ/AZ) will require even more rigorous monitoring for signs of cardiotoxicity. Both medications have been independently shown to increase the risk of QTc-prolongation, drug-induced *torsade de pointes* and drug-induced sudden cardiac death.

The South African Health Products Regulatory Authority (SAHPRA) has recently cautioned against the stockpiling of medicines like CQ because the management of the current coronavirus-related pandemic, as well as the potential benefits and risks of the various treatment options being proposed, are still under investigation.^21^

### Important to note in terms of chloroquine salt versus chloroquine base

The locally available products are presented in either tablet or capsule form, containing 200 mg of CQ sulphate (salt form). Per the manufacturers, this translates into 146.7 mg of CQ base per tablet. In the case of CQ phosphate, 250 mg will be equivalent to approximately 150 mg of the CQ base. Always check the manufacturers’ package inserts to confirm the applicable base dosages.

### Why knowing this is important

Chloroquine becomes toxic at high dosage levels that exceed 1000 mg per day; thus, it is important to ensure strict monitoring because the recommended dosages to be used in patients with COVID-19 are dangerously close to toxic levels.

To convert the required dosage given as CQ base, to the CQ sulphate salt equivalent, divide by a factor of 0.73 (e.g. 146 mg base ÷ 0.73 = 200 mg CQ sulphate to be dosed).

## Emerging data and evolving treatment options in COVID-19

Research in this field is currently ongoing. As new data become available, guidelines and recommendations may need to be updated accordingly.

At present, no clinically approved antiviral or immunomodulatory treatment options or preventative vaccines exist for COVID-19and those that are being considered are still under investigation, with a small number of patients already enrolled in clinical trials.^2,14,15,18^

Several existing antiviral agents are being used under clinical trial and compassionate use protocols based on their *in vitro* activity (against this or other related viruses) and based on limited clinical experience.^22^

Table 3 summarises the current therapies of concern in patients with COVID-19. Table 4 includes a broad overview of some of the investigational (or experimental) treatment options that are currently being studied in the management of COVID-19.

**TABLE 3:** Therapies of concern in COVID-19 patients.^4,14,23,24,25^

Therapeutic agents of concern	Current information and guidance
Non-steroidal anti-inflammatory drugs (NSAIDs)^4,14,23,24,25^	The use of ibuprofen (or other NSAIDs) in COVID-19 patients has been raised as a potential concern. The virus that causes COVID-19 binds to its target cells via angiotensin-converting enzyme 2 (ACE2), which is expressed by the epithelial lining of blood vessels, the kidneys, the intestines, and the lungs. Ibuprofen is one of the drugs that increases ACE2 expression through upregulation, which could theoretically accelerate COVID-19 infection. As yet, there is no scientific evidence to support the complete dismissal of the drug in COVID-19 patients. However, the current recommendation is to strongly favour the use of paracetamol in the management of COVID-19 related pain and fever, to be used at the recommended dosage levels to avoid any possible liver damage.
Angiotensin-converting enzyme inhibitors (ACE-inhibitors) and angiotensin receptor blockers (ARBs)^4,14^	Recent work suggests that patients on angiotensin-converting enzyme (ACE) inhibitors or angiotensin receptor blockers (ARBs), as well as the thiazolidinediones, as part of their treatment regimen for hypertension, heart disease and/or diabetes mellitus, may also display an upregulation of ACE2. This might place these patients at risk of worse outcomes when they contract COVID-19. For the moment, this remains theoretical, with no direct evidence of a linkage to poor clinical outcomes. In addition, discontinuing or switching such patients’ ACE-inhibitor or ARB therapy to alternative agents may be deleterious to their care. Pending further evidence, it is therefore not recommended that patients be switched from their ACE-inhibitors or ARBs, unless there are other medical reasons for doing so.

**TABLE 4:** Treatment options under investigation for use in patients with COVID-19.^1,5,14,15,17,18,19,22,26,17,18,19,30,31^

Agents	Variable
**Existing antiviral agents**	
Lopinavir/ritonavir (LPV/r)^5,15,17,18,26^	This is an approved combination antiretroviral treatment for HIV infection. Lopinavir and ritonavir are both protease-inhibitors that block viral replication. LPV/r has been used for other coronavirus infections in the past and is being investigated in multiple randomised controlled trials in China. However, in one such randomised, controlled, open-label study that involved confirmed and hospitalised SARS-CoV-2 infected adult patients, which were assigned in a 1:1 ratio to either receive LPV/r in addition to their standard of care, or standard of care only, it was concluded that treatment with LPV/r was not associated with a difference from standard of care (i.e. the time to clinical improvement and the 28-day mortality rate was essentially similar in both groups). However, the researchers did suggest that their early data should inform future studies accordingly. Because a possible benefit has been suggested (i.e. a shorter stay in the intensive care unit in patients that received LPV/r before the 12th day from the onset of their symptoms), LPV/r may still be considered as a possible second choice.
Oseltamivir^17,22^	Without knowing which specific treatment options are effective, it is risky to prescribe an influenza treatment like oseltamivir. Oseltamivir is not effective against COVID-19. However, if used as initial empiric treatment during the influenza season, oseltamivir could be a rational choice in a critically ill patient when there is a high suspicion of influenza-related pneumonia.
**Other agents**	
Ascorbic acid (vitamin C)^17^	The use of vitamin C supplementation in viral pneumonia is currently not supported by high-quality evidence, although limited evidence in animal models of coronavirus suggest that it could have some beneficial effects.
Corticosteroids^1,14,15,31^	The routine use of corticosteroids in COVID-19 patients is not recommended unless clinically indicated for another reason, such as chronic obstructive pulmonary disease (COPD) for example.^1,22^ Corticosteroids are therefore not indicated in treating SARS-CoV-2 as per the available evidence, because they may prolong viral shedding.
Sarilumab^29^	Sarilumab is a rheumatoid arthritis agent, being a fully human monoclonal antibody, which inhibits the interleukin-6 (IL-6) pathway and is used as part of a clinical programme to treat hospitalised patients with severe COVID-19 coronavirus infection. IL-6 is associated with the overactive inflammatory response in the lungs of severely or critically ill COVID-19 patients. This agent is not yet available in SA.
Tocilizumab^18,19,31^	An IL-6 inhibitor used for moderate to severe active rheumatoid arthritis that is not responding to other therapies. Proposed to reduce the cytokine storm in COVID-19. Adverse effects: elevation of liver enzymes and an increased risk of reactivation of other respiratory infections.
**Novel antiviral agents**	
Remdesivir^15,27^	Clinical trials are underway to investigate this antiviral agent for the treatment of COVID-19 in adults. It has significant *in vitro* activity against coronaviruses and some evidence of efficacy in an animal model of MERS. Remdesivir has been administered to several hundred patients with confirmed, severe SARS-CoV-2 infections in North America, Europe and Japan through the use of expanded access or compassionate use programmes, because it has not completed clinical development yet. Similar early access programmes are to be made available in other countries as well. Remdesivir treatment has been shown to shorten the time to recovery in some patients with COVID-19. Remdesivir is not yet available in South Africa, except via a possible Section 21 authorisation.
Sofosbuvir (in combination with ribavirin)^28^	Data from a molecular docking experiment using the SARS-CoV-2 RNA-dependent RNA polymerase (RdRp) model identified tight binding of sofosbuvir and ribavirin to the coronavirus RdRp, thereby suggesting possible efficacy of sofosbuvir and ribavirin in treating the COVID-19 infection. Sofosbuvir is not yet available in South Africa.
Favipiravir^30^	Favipiravir is a drug used to treat new strains of influenza and appears to have efficacy in patients with coronavirus. Favipiravir is not yet available in South Africa.

RNA, Ribonucleic acid.

## The approach of the pharmacist towards suspected or confirmed COVID-19 patients

It is widely accepted that pharmacists play a vital role as a first point of contact within many healthcare systems.^1^ Thus, pharmacists can contribute to the containment of a global pandemic such as COVID-19.

To reduce and prevent transmission during this pandemic, pharmacists should ensure that the following measures are in place within their community or institutional pharmacies:

- Safe social (and professional) distancing of at least 1 metre between queueing patients (the use of floor markings is recommended) and between pharmacy personnel and patients across the counter
- Appropriate respiratory hygiene (which includes the appropriate use of personal protective equipment (PPE) amongst staff and patients
- Effective hand hygiene amongst staff and patients throughout the day
- Surfaces within the pharmacy wiped down on a regular basis
- Staff members encouraged to stay at home when feeling unwell.

Furthermore, it is vital not to lose sight of the importance of the full list of essential medicines that should ordinarily be available in healthcare facilities, whether or not patients with suspected or confirmed COVID-19 are being managed there. Priorities need to be reassessed continuously and emergency procurement procedures should be instituted to ensure faster and easier access to stock. Inpatient facilities need to review the stocking levels of ward or unit supplies, and the pharmacist should endeavour to assist in procuring stock of experimental, clinical trial or Section 21-based supplies as needed.

In addition to these measures, the scenarios outlined in Table 5 will help to guide the interaction between the community or institutional pharmacist and a suspected or confirmed COVID-19 patient.

**TABLE 5:** Scenarios to guide the interaction between the community or institutional pharmacist and a suspected or confirmed COVID-19 patient.^32,33,34^

Scenario 1: Community Pharmacy: Patients call or walk into the pharmacy with symptoms of COVID-19 or suspect that they may have COVID-19	Scenario 2: Patients with symptoms of COVID-19 or that suspect they may have COVID-19, present to a hospital or clinic	Scenario 3: A confirmed COVID-19 patient who has commenced self-isolation at home and has been supplied with medication. The information below can be communicated telephonically or in writing when the medication is being collected by a family member, friend or other
Maintain social distancing whilst consulting with the patient.	These patients would have minimum contact with the pharmacist. The attending doctor or nurse should ensure the following:	Medication counselling: confirm and check the dosage, check for any allergies or contra-indications. Counsel the patient on when to take medication, how often, and caution them regarding any possible side effects.
Confirm information relating to recent travel history or contact with persons who had recently travelled to countries with COVID-19 outbreaks, or personal contact with a person with confirmed COVID-19, or symptoms associated with COVID-19 that he or she may be experiencing.	Assessment of the patient and information obtained from the patient pertaining to recent travel and possible contact with others.	Emphasise self-isolation: that they must not leave their home to go to any public places.
Patients that are symptomatic (e.g. coughing) must wear a mask to reduce the risk of spreading any infection.	Contact with hospital staff is restricted and the patient confined to a specific area of the facility to reduce the risk of spreading the virus.	Avoid or keep any contact to a minimum (avoid same rooms and maintain two metre distancing) with family members that reside at the same home. Discourage any visitors.
Advise the patient to contact their doctor or clinic for assessment and referral to a pathology laboratory for testing. It is recommended to phone before going to the doctor or clinic to minimise unnecessary contact. This will also allow staff to take necessary safety precautions to assist the patient promptly whilst safeguarding themselves and others at the facility from possible infection.	Swabbed patients with mild to moderate symptoms (but clinically well) are required to self-isolate at home until the results have been received, and after the clinician’s assessment.	Ensure good ventilation of the room where a confirmed COVID-19 patient is being isolated, with open windows, if possible.
Inform the patient about the expected process of examination and swabbing by the doctor, if asked to attend a consultation.	Patients with severe symptoms will be admitted to the respective or designated hospital or facility.	Advise the patient (and stay-in family) to maintain proper hand hygiene by washing their hands with soap and hot water for 20 s or use an alcohol-based hand sanitiser.
Advise the patient to self-isolate at home until the test results are known, or as advised by the doctor.	Patients are immediately isolated in the admitting ward.	Avoid touching the face, mouth, nose and eyes.
Advise the patient to maintain proper hand hygiene by washing their hands with soap and hot water for 20 s or use an alcohol-based hand sanitiser.	Doctors, nurses, pharmacists and support staff caring for the patient reduce the risk of spreading the virus by limiting access to the patient’s room, maintaining strict hand and respiratory hygiene, and ensuring the correct use of personal protective equipment (i.e. gowns, mask, gloves, etc.).	Practise respiratory hygiene: Advise the patient to wear a suitable face mask (fit and dispose of the mask correctly to offer effective protection against the spread of infection). In addition, if the patient has a cough, he or she should cover the mouth and nose with a bent elbow or tissue.
Provision of over the counter (OTC) medication for relief of mild to moderate symptoms, e.g. paracetamol, mucolytics etc.	Review and dispensing of prescribed medication.	Dispose of tissues immediately after use in a separate rubbish bag.
Contact the doctor if symptoms worsen.	All wards/treating areas are to be thoroughly disinfected once patients are moved or discharged.	Advise the patient on the use of separate towels, disposable crockery and utensils, or wash separately with hot water and detergent.
Disinfect countertop surfaces when patient has left the pharmacy and ensure proper hand hygiene.		Disinfect all surfaces touched by the patient with a bleach-based disinfectant after use or contact, especially in shared bathrooms.
		Contact the doctor if symptoms worsen.

## Conclusion

In record time, COVID-19 has become a truly debilitating adversary the world over. Pharmacists find themselves on the frontline of the fight against this disease. It is thus of paramount importance that all pharmacy personnel follow strict infection control guidelines and make use of personal protective gear at all times to protect both themselves and their patients. Severe illness is likely to strike the same population groups that are at high risk for developing complications as well. COVID-19, therefore, also provides a rare and unique opportunity for pharmacists to gain first-hand experience in dealing with pandemics of this nature and will also serve to draw attention to other relevant healthcare issues, such as the importance of vaccination against childhood illnesses, as well as seasonal influenza.
